# Efficacy and Safety of EGFR Tyrosine Kinase Inhibitors Combined with Cranial Radiotherapy for Brain Metastases from Non-Small-Cell Lung Cancer: A Protocol for a Systematic Review and Meta-Analysis

**DOI:** 10.1155/2022/6531748

**Published:** 2022-07-13

**Authors:** Yuansha Ge, Bowen Xu, Heping Wang, Junmao Gao, Xiaoxiao Zhang, Taicheng Lu, Ruike Gao, Jie Li

**Affiliations:** ^1^Department of Oncology, Guang'anmen Hospital, China Academy of Chinese Medical Sciences, Beijing, China; ^2^Beijing University of Chinese Medicine, Beijing, China; ^3^China Academy of Chinese Medical Sciences, Beijing, China; ^4^Department of Radiology, Guang'anmen Hospital, China Academy of Chinese Medical Sciences, Beijing, China

## Abstract

*Introduction*. Brain metastases (BMs) are common in non-small-cell lung cancer (NSCLC), which leads to a poor prognosis. As the two most effective strategies available, the use of combination of epidermal growth factor receptor tyrosine kinase inhibitors (EGFR-TKIs) and radiotherapy (RT) is still controversial. This protocol proposes a methodology for carrying out a systematic review and meta-analysis that is aimed at (1) focusing on the efficacy and safety role of EGFR-TKIs combined with RT for BMs from NSCLC and (2) displaying the difference in efficacy of EGFR-TKIs owing to the sites and number of BMs, different types of RT, EGFR mutation status, and the subtypes of EGFR mutations by subgroup analysis. *Methods and Analysis*. Electronic databases including PubMed, Embase, CENTRAL, Web of Science, CBM, CNKI, Wanfang database, and VIP database will be searched from their inception until May 2022. Only randomized controlled trials evaluating the clinical efficacy and safety of EGFR-TKIs combined with RT on BMs of NSCLC will be included. Two reviewers will select the articles, assess the risk of bias, and extract data independently and in duplicate. The RoB 2 tool will be used to assess the quality of included studies. The meta-analysis of data synthesis will be performed with Stata 16. Publication bias will be assessed with the funnel plot method and the Egger test. Quality of the evidence will be evaluated by the GRADE system. *Discussion*. The approval of an ethical committee is not required. All the included trials will comply with the current ethical standards and the Declaration of Helsinki. Given the ongoing controversies regarding the optimal sequencing of the available and expanding treatment options for EGFR-TKIs in NSCLC with BMs, a synthesis of available, high-quality clinical research evidence is essential to advance our understanding in the treatment of this complex and common disease. This systematic review will evaluate available evidence, will try to provide optimized advice in the applications of EGFR-TKIs, and will be published in a high-quality journal. This study is registered with PROSPERO registration number CRD42021291509.

## 1. Introduction

According to GLOBOCAN 2020, there were 2,206,771 new cases (11.4% of total new cancer cases) and 1,796,144 new deaths (18.0% of total cancer deaths) of lung cancer worldwide in 2020, ranking second in the incidence and first in the mortality of malignant tumors [1]. Non-small-cell lung cancer (NSCLC) accounts for 85% of all lung cancers [2]. One of the most common distant metastatic sites of NSCLC is the brain, approximately 30-50% of patients will develop brain metastases (BMs) during the course of their disease [3, 4], and patients with BMs have a poor prognosis with median survival ranging from 3 to 14 months [5].

Radiotherapy (RT) such as whole-brain radiotherapy (WBRT), stereotactic radiosurgery (SRS), or stereotactic radiotherapy (SRT) is a primary treatment for patients with BMs [6]. SRS and SRT are suitable for selected patients with a limited number of BMs [7]. Multiple trials have shown their survival benefit [8–11] and improvement of local control [12] of BMs, while WBRT is commonly used in patients with multiple brain lesions or diffuse BMs who are not suitable for SRS [6]. But data on the overall survival (OS) benefit of cranial RT are conflicting [13].

Over the past decades, the advent of novel systemic therapies, including targeted therapy, has revolutionized therapy for NSCLC. On the one hand, a prospective, multinational, epidemiological study has shown that approximately 51.4% of Asian lung adenocarcinoma patients were with the epidermal growth factor receptor (EGFR) activating mutations [14]. EGFR tyrosine kinase inhibitors (EGFR-TKIs) have played a significant role in the treatment of NSCLC [15]. On the other hand, growing evidence suggests that BMs occur most frequently in NSCLC patients with adenocarcinomas and tumors harboring EGFR mutations [16]. For NSCLC patients with BMs, who have EGFR mutations, the standard therapy includes EGFR-TKIs, such as osimertinib, afatinib, gefitinib, erlotinib, and icotinib [17]. Also, TKIs including erlotinib and gefitinib have been demonstrated that can penetrate the blood-brain barrier [6, 18], and a new study even showed that third-generation EGFR-TKIs like osimertinib have the higher brain penetrance [19]. The research showed that using EGFR-TKIs can reduce the risk of BMs compared with chemotherapy [20]. Several phase III trials including patients with NSCLC and BMs demonstrated that EGFR-TKIs were associated with a higher progression-free survival (PFS) compared to chemotherapy [21, 22].

As the two most effective interventions available, EGFR-TKIs and RT have synergistic effects [23–26], and the combination of these two interventions might be expected to improve the control of BMs. However, the use of EGFR-TKIs with RT is still the most contentious area.

Two previous phase II trials of NSCLC patients with BMs have shown that erlotinib combined with WBRT demonstrated a favorable objective response rate and improved the median OS and median central nervous system PFS (CNS-PFS) in patients with BMs of NSCLC [26, 27].

Also, there were three meta-analyses to intuitively synthesize the clinical study evidence of EGFR-TKIs with or without cranial RT. Two systematic reviews demonstrated that cranial RT with EGFR-TKIs might improve survival outcomes [28, 29]. Contrary to clinical routine knowledge, one of the systematic reviews also suggests that the combination group could reduce the incidence of myelosuppression (III-IV) [29]. However, these two articles included noncomparative studies or case-control studies, and the evidence's quality was not high [28, 29].

A 2020 newly published head-to-head evidence comparison of TKIs with or without RT in EGFR mutation NSCLC presents inconsistent treatment options. In contrast, this study reflected the short-term benefits of EGFR-TKIs but did not translate into long-term survival benefits, while combination therapy might have more advantages in long-term survival [30]. In the meanwhile, a recently completed multicenter, open-label, randomized, controlled phase III trial indicated that concurrent erlotinib with WBRT failed to show a significant improvement in CNS-PFS, PFS, and OS, compared with WBRT alone. Also, in the EGFR-mutant subgroup, WBRT with erlotinib did not significantly improve the CNS-PFS over the WBRT-alone arm, nor was the PFS or OS improved. However, it showed that both interventions were safe and well tolerated [31]. This study demonstrated that it is inconsistent with the results of previous phase II trials and meta-analysis, so it also gives us more thinking about EGFR-TKIs combined with RT.

Based on the findings, we believe that exploring the new clinical study evidence of EGFR-TKIs combined with RT and finding their role in NSCLS patients with BMs are essential.

## 2. Objectives

This protocol proposes a methodology for carrying out a systematic review and meta-analysis that is aimed at (1) focusing on the efficacy and safety role of EGFR-TKIs combined with RT for BMs from NSCLC and (2) displaying the difference in efficacy of EGFR-TKIs owing to the sites of BMs, the numbers of BMs, different types of RT, EGFR mutation status, and the subtypes of EGFR mutations by subgroup analysis.

## 3. Materials and Methods

### 3.1. Study Registration

This systematic review will be guided by the Preferred Reporting Items for Systematic Reviews and Meta-Analyses (PRISMA) statement [32]. This study has been registered on PROSPERO (CRD42021291509). This protocol was conducted according to the PRISMA Protocols (PRISMA-P) statement [33]. The PRISMA-P checklist is available as Additional file 1.

### 3.2. Eligibility Criteria

#### 3.2.1. Participants

This study will review RCTs, including participants with BMs of NSCLC diagnosed through the pathological and radiographical tests.

#### 3.2.2. Interventions and Controls

The treatments administered to the control group will be cranial RT as the sole treatment, while the intervention group will receive EGFR-TKI combined with cranial RT (WBRT, SRS, or SRT).

#### 3.2.3. Outcomes

This study will include RCTs reporting the clinical efficacy of HM. Studies reporting only the outcomes of laboratory tests would be excluded.

#### 3.2.4. Study Type

This systematic review will include RCTs. Observational studies and animal studies will not be included.

### 3.3. Outcomes of Interest

The outcomes evaluated in this study will include those related to clinical efficacy and safety. The primary outcomes will include overall response rate (ORR) and overall survival (OS). Response rates calculated using the Response Evaluation Criteria in Solid Tumors (RECIST) [34] or WHO criteria [35] will be included. Given the strong correlation between these two antitumor treatment response evaluation criteria, the outcomes reported by them were considered homogeneous and pooled together [36]. Secondary outcomes will include progression-free survival (PFS), central nervous system progression-free survival (CNS-PFS), DCR, remission rate of the central nervous system (RR-CNS), 1-year survival rate, mortality rate, and quality of life. Safety outcomes will focus on the incidence of AEs, including neurological AEs such as tremors, vertigo, and dizziness, based on the WHO criteria [35] or the National Cancer Institute Common Terminology Criteria for Adverse Events (CTCAE) [37].

### 3.4. Search Strategy

An electronic database search will include PubMed, Embase, CENTRAL, Web of Science, Chinese Biomedical Literature Database (CBM), China National Knowledge Infrastructure (CNKI), Wanfang database, and Chinese Scientific Journals Database (VIP database). The databases will be searched from their inception until May 2022, and we plan to update our search prior to submission of the full report for publication. The language will be restricted to English and Chinese. And we will search the Chinese Clinical Trial Registry (ChiCTR) and ClinicalTrials.gov to identify ongoing and completed trials.

The search strategy will be developed from the combination of controlled vocabulary (MeSH terms, Emtree terms) and free-text terms. The MeSH terms of “Cranial Irradiation”, “Carcinoma, Non-Small-Cell Lung”, “Gefitinib”, “Erlotinib Hydrochloride”, “icotinib”, “Afatinib”, “dacomitinib”, and “osimertinib” and relevant entry terms will be used in the construction of a search strategy of PubMed, which is shown in Additional file 2. Modifications to this search strategy will be used with other databases.

One author (BX) will be responsible for a manual search, which will involve cross-checking the references of all relevant systematic reviews to obtain additional studies.

The process of screening and selection, data extraction, and risk of bias assessment will be done by two review members (BX and YG) independently and in duplicate. Any disagreements will be solved by discussion or the interposition of another review member (HW).

### 3.5. Screening and Selection

**Figure 1:**
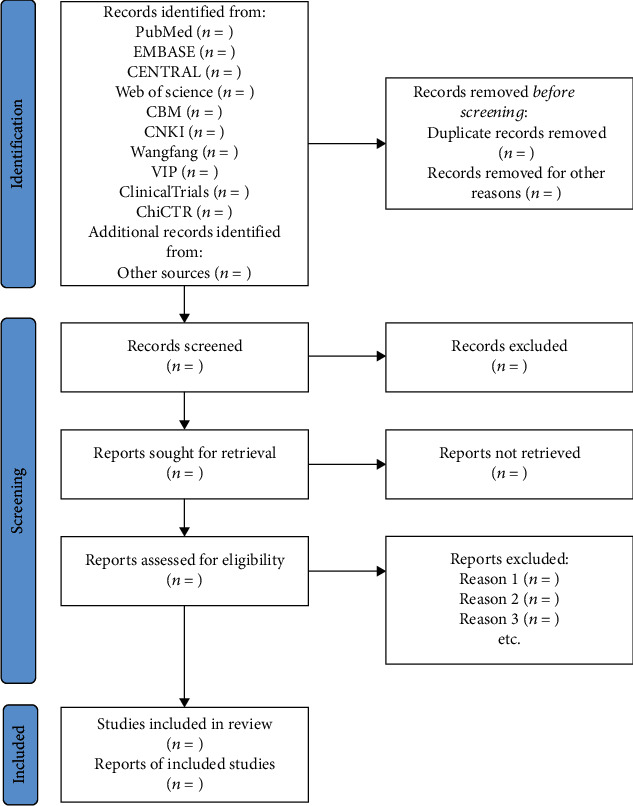
Flowchart of study selection.

Figure 1 presents a flowchart of the screening and selection process. Search results will be imported to EndNote 20. Two review authors (BX and YG) will access titles and abstracts from the results of the database search after removal of duplication. After that, full text will be reviewed and assessed in terms of eligibility. RCTs that met the eligibility criteria will be included. The process will be summarized using a PRISMA flow diagram in the full report [32].

### 3.6. Data Extraction

Microsoft Excel will be used in the process of data extraction. We will contact researchers of original studies for missing or incomplete data if necessary. The following data will be extracted from included studies: (1) identification information (first author, year of publication), (2) general information (study location, study setting (single-center or multicenter; blinded or unblinded), sample size, duration of follow-up, and funding source), (3) participants (age, gender, race, EGFR mutation status, site of BMs, and number of BMs), (4) intervention details (name and generation of EGFR-TKI, dose of the treatment, and duration of the treatment), (5) comparison details (type of RT, dosage of RT), and (6) outcome details (clinical outcomes and their results).

### 3.7. Quality Assessment

The Cochrane Collaboration's Risk of Bias 2 (RoB 2) tool will be used to assess the methodological quality of included studies [38]. We will evaluate each study of randomization process, deviations from intended interventions, missing outcome data, measurement of the outcome, and selection of the reported result. The risk of bias of individual studies will be assessed as low, some concerns, and high risk of bias.

### 3.8. Evidence Synthesis for RCTs

The meta-analysis will be carried out if adequate data of primary or secondary outcomes are obtainable and the results among the studies are homogeneous, and forest plots will be presented. The risk ratio (RR) for dichotomous data and mean differences (MD) for continuous data with 95% confidence intervals (CIs) will be evaluated. The random effects model will be used when clinical or statistical heterogeneity exists; otherwise, the fixed effects model will be used in the data synthesis. We will quantify statistical inconsistency by applying the *I*^2^ statistic; a value of *I*^2^ > 50% and >75% will indicate substantial and considerable heterogeneity, respectively [39]. The subgroup analysis and sensitivity analysis will be performed to explore the source of heterogeneity. Stata 16 will be used in the data synthesis.

Meta-analysis may be precluded in some conditions (e.g., limited evidence for comparison, incompletely reported outcome, different effect measures, and statistical heterogeneity), and descriptive analysis will be performed in these conditions [39].

### 3.9. Publication Bias

The publication bias of the cumulative evidence among individual studies will be evaluated by a graphical method of the funnel plot and the Egger test [40], if at least ten studies are included for the synthesized outcome.

### 3.10. Additional Analysis

#### 3.10.1. Subgroup Analysis

When conducting a meta-analysis, several subgroup analyses will be performed to identify subpopulations that may be associated with the difference in EGFR-TKI efficacy. The subgroup analyses will be performed according to (1) the race and gender of participants, (2) the status and subtypes of EGFR mutations, (3) the generation and name of EGFR-TKIs, (4) whether the participants have received the EGFR-TKI treatment previously, and (5) the site and number of BMs.

#### 3.10.2. Sensitivity Analysis

To assess the stability of results, sensitivity analyses, by exclusion of each type of RT, generations of EGFR-TKIs, and risk of bias, were performed for ORR and RR-CNS outcomes.

### 3.11. Quality of Evidence

The quality of the cumulative evidence will be evaluated by the Grading of Recommendations Assessment, Development, and Evaluation (GRADE) system. Risk of bias of included studies, inconsistency, indirectness, imprecision, and reporting bias will be assessed to determine whether the certainty of evidence should be downgraded. The quality of evidence will be classified as high, moderate, low, or very low [41].

## 4. Discussion

Approximately 10-25% of NSCLC patients present BMs at the time of diagnosis, and 25% to 50% develop BMs during the course of the disease, which remained the leading cause of death in NSCLC patients [42, 43]. EGFR-TKIs and RT, as the two most effective strategies available, which are often used sequentially or in combination to gain synergistic effects might be expected to improve the control of BMs from NSCLC [44]. However, given the ongoing controversies regarding the optimal sequencing of the available and expanding treatment options for NSCLC with BMs [45], synthesis of the best available clinical evidence is essential to advance our understanding of this complex and common disease.

Previous clinical trials and meta-analyses have demonstrated that EGFR-TKIs combined with RT can effectively improve CNS-PFS, PFS, OS, ORR, DCR, and survival rate [26–29]. With the publication of the latest phase III clinical trial [31], the results of the previous researches have been impacted, which requests further research about EGFR-TKIs combined with RT. In addition, the literature retrieval time of the latest meta-analysis was up to November 2020 [30], not to mention the unfavorable quality of evidence, the absence of GRADE evaluation, and the unconfirmed prioritization of EGFR-TKIs with or without RT for BMs of NSCLC.

Interestingly, a retrospective study found that patients with BMs from NSCLC with different EGFR mutation subtypes (exon 21 and exon 19) will experience different clinical outcomes in EGFR-TKIs alone and EGFR-TKIs combined with RT [46]. This suggested that more advantageous treatment options can be selected according to EGFR mutation subtypes and also further emphasizes the personalized treatment of precision medicine.

Therefore, in view of the inconclusive data published until now, we believe that the previous recalcitrant cognition may be broken with the publication of high-quality clinical researches and the ongoing prospective clinical trials [47].

In this study, we will make available the following two key scientific clinical questions: (1) how is the efficacy and safety of EGFR-TKIs combined with RT in patients with BMs from NSCLC and (2) to what extent do patients benefit from combination therapy. First, we will compare the efficacy (OS, PFS, CNS-PFS, ORR, and DCR) and safety (grade 3 or higher adverse events) of RT with or without EGFR-TKIs. Second, we will evaluate differences in the efficacy of combination therapy in several different populations and explore more precisely the benefit population. The correlation between EGFR mutant population, the subtypes of EGFR mutations, the sites of BMs, the number of BMs, and race and gender will be displayed by subgroup analysis. Any other outcomes reported in the eligible studies will be extracted and reported, such as cognitive function.

This study will yield valuable treatment regimen guidance for patients with EGFR mutation NSCLC and give more precise guidance of clinical treatment application. However, some potential literature (such as grey literature) may not be included since the literature search strategy is limited, and thus, our findings may be altered.

We also hope to use data from upcoming studies to update existing evidence.

## Data Availability

No data were used to support this study.

## Abbreviations

BM: Brain metastasis
EGFR: Epidermal growth factor receptor
TKI: Tyrosine kinase inhibitor
RT: Radiotherapy
WBRT: Whole-brain radiation therapy
SRS: Stereotactic radiosurgery
SRT: Stereotactic radiotherapy
RoB: Risk of bias
CI: Confidence interval
GRADE: Grading of Recommendations Assessment, Development, and Evaluation
RR: Risk ratio
MD: Mean difference
OS: Overall survival
ORR: Overall response rate
DFS: Disease-free survival
PFS: Progress-free survival
CNS-PFS: Central nervous system progress-free survival
RR-CNS: Remission rate of the central nervous system
DCR: Disease control rate
AE: Adverse events
RCT: Randomized controlled trial
RECIST: Response Evaluation Criteria in Solid Tumors
CTCAE: Common Terminology Criteria for Adverse Events.

## Supplementary Materials

Supplementary 1 Additional file 1: PRISMA-P checklist.

Supplementary 2 Additional file 2: search strategy for PubMed.
