# Supplementary material for: Efficacy and Safety of EGFR Tyrosine Kinase Inhibitors Combined with Cranial Radiotherapy for Brain Metastases from Non-Small-Cell Lung Cancer: A Protocol for a Systematic Review and Meta-Analysis
Source: Biomed Res Int. 2022 Jul 13;2022:6531748. doi: 10.1155/2022/6531748 (PMC9301690; doi:10.1155/2022/6531748)
Supplement: Supplementary 2 — Additional file 2: search strategy for PubMed. [file 6531748.f2.docx]

**Search strategy used in PubMed**

#1 (("Carcinoma, Non-Small-Cell Lung"[Mesh]) OR (((((((((((Carcinoma, Non Small Cell Lung[Title/Abstract]) OR (Carcinomas, Non-Small-Cell Lung[Title/Abstract])) OR (Lung Carcinoma, Non-Small-Cell[Title/Abstract])) OR (Lung Carcinomas, Non-Small-Cell[Title/Abstract])) OR (Non-Small-Cell Lung Carcinomas[Title/Abstract])) OR (Non-Small-Cell Lung Carcinoma[Title/Abstract])) OR (Non Small Cell Lung Carcinoma[Title/Abstract])) OR (Carcinoma, Non-Small Cell Lung[Title/Abstract])) OR (Non-Small Cell Lung Carcinoma[Title/Abstract])) OR (Non-Small Cell Lung Cancer[Title/Abstract])) OR (Nonsmall Cell Lung Cancer[Title/Abstract])))

#2 ((((((((((Brain Metastases[Title/Abstract]) OR (Brain Metasta[Title/Abstract])) OR (Limited brain metastases[Title/Abstract])) OR (Extensive brain metastases[Title/Abstract])) OR (Brain lesions[Title/Abstract])) OR (CNS Metastases[Title/Abstract])) OR (Central Nervous system Metastases[Title/Abstract])) OR (Nervous system Metastases[Title/Abstract])) OR (multiple Metastases lesions[Title/Abstract])) OR (multiple-Metastases[Title/Abstract]))

#3 (((((((((((((((epidermal growth factor receptor-tyrosine kinase inhibitor[Title/Abstract]) OR (EGFR-TKIs[Title/Abstract])) OR (EGFR-TKI[Title/Abstract])) OR (((osimertinib[Supplementary Concept]) OR (tagrisso[Title/Abstract])) OR (AZD9291[Title/Abstract]))) OR ("Erlotinib Hydrochloride"[Mesh])) OR ((((((((((((((((((Hydrochloride, Erlotinib[Title/Abstract]) OR (Erlotinib HCl[Title/Abstract])) OR (HCl, Erlotinib[Title/Abstract])) OR (OSI-774[Title/Abstract])) OR (OSI 774[Title/Abstract])) OR (OSI774[Title/Abstract])) OR (CP 358774[Title/Abstract])) OR (358774, CP[Title/Abstract])) OR (CP 358,774[Title/Abstract])) OR (358,774, CP[Title/Abstract])) OR (CP-358,774[Title/Abstract])) OR (CP358,774[Title/Abstract])) OR (CP-358774[Title/Abstract])) OR (CP358774[Title/Abstract])) OR (11C-erlotinib[Title/Abstract])) OR (11C erlotinib[Title/Abstract])) OR (Erlotinib[Title/Abstract])) OR (Tarceva[Title/Abstract]))) OR ("Afatinib"[Mesh])) OR (((((((((((BIBW-2992-MA2[Title/Abstract]) OR (BIBW 2992 MA2[Title/Abstract])) OR (BIBW-2992MA2[Title/Abstract])) OR (BIBW 2992MA2[Title/Abstract])) OR (BIBW2992 MA2[Title/Abstract])) OR (Afatinib Maleate[Title/Abstract])) OR (BIBW 2992[Title/Abstract])) OR (BIBW2992[Title/Abstract])) OR (BIBW-2992[Title/Abstract])) OR (Gilotrif[Title/Abstract])) OR (Afatinib Dimaleate[Title/Abstract]))) OR ("Gefitinib"[Mesh])) OR (((Iressa[Title/Abstract]) AND (ZD1839[Title/Abstract])) AND (ZD 1839[Title/Abstract]))) OR ("dacomitinib" [Supplementary Concept])) OR ((((Vizimpro[Title/Abstract]) OR (PF 00299804[Title/Abstract])) OR (PF00299804[Title/Abstract])) OR (PF-00299804[Title/Abstract]))) OR ("icotinib" [Supplementary Concept])) OR ("aumolertinib" [Supplementary Concept])) OR (((almonertinib[Title/Abstract]) OR (HS-10296[Title/Abstract])) OR (furmonertinib[Title/Abstract])))

#4 ((("Radiotherapy"[Mesh]) OR ((((((((((((((((((Radiotherapies[Title/Abstract]) OR (Radiation Therapy[Title/Abstract])) OR (Radiation Therapies[Title/Abstract])) OR (Therapies, Radiation[Title/Abstract])) OR (Therapy, Radiation[Title/Abstract])) OR (Radiation Treatment[Title/Abstract])) OR (Radiation Treatments[Title/Abstract])) OR (Treatment, Radiation[Title/Abstract])) OR (Radiotherapy, Targeted[Title/Abstract])) OR (Radiotherapies, Targeted[Title/Abstract])) OR (Targeted Radiotherapies[Title/Abstract])) OR (Targeted Radiotherapy[Title/Abstract])) OR (Targeted Radiation Therapy[Title/Abstract])) OR (Radiation Therapies, Targeted[Title/Abstract])) OR (Targeted Radiation Therapies[Title/Abstract])) OR (Therapies, Targeted Radiation[Title/Abstract])) OR (Therapy, Targeted Radiation[Title/Abstract])) OR (Radiation Therapy, Targeted[Title/Abstract]))) OR ((((((Whole brain radiotherapy[Title/Abstract]) OR (WBRT[Title/Abstract])) OR (Stereotactic Radiosurgery[Title/Abstract])) OR (SRS[Title/Abstract])) OR (Stereotactic radiotherapy[Title/Abstract])) OR (SRT[Title/Abstract])))

#5 ((randomized controlled trial[pt] OR controlled clinical trial[pt] OR randomized[tiab] OR placebo[tiab] OR clinical trials as topic[mesh:noexp] OR randomly[tiab] OR trial[ti] NOT (animals[mh] NOT humans [mh])))

#6 #1 AND #2 AND #3 AND #4 AND #5
